# NSMAP: A method for spliced isoforms identification and quantification from RNA-Seq

**DOI:** 10.1186/1471-2105-12-162

**Published:** 2011-05-16

**Authors:** Zheng Xia, Jianguo Wen, Chung-Che Chang, Xiaobo Zhou

**Affiliations:** 1 Department of Radiology, The Methodist Hospital Research Institute, Houston, TX 77030, USA; 2 Department of Pathology, The Methodist Hospital Research Institute, Houston, TX 77030, USA; 3 Weill Cornell Medical College, New York, NY 10065, USA

## Abstract

**Background:**

The development of techniques for sequencing the messenger RNA (RNA-Seq) enables it to study the biological mechanisms such as alternative splicing and gene expression regulation more deeply and accurately. Most existing methods employ RNA-Seq to quantify the expression levels of already annotated isoforms from the reference genome. However, the current reference genome is very incomplete due to the complexity of the transcriptome which hiders the comprehensive investigation of transcriptome using RNA-Seq. Novel study on isoform inference and estimation purely from RNA-Seq without annotation information is desirable.

**Results:**

A Nonnegativity and Sparsity constrained Maximum APosteriori (NSMAP) model has been proposed to estimate the expression levels of isoforms from RNA-Seq data without the annotation information. In contrast to previous methods, NSMAP performs identification of the structures of expressed isoforms and estimation of the expression levels of those expressed isoforms simultaneously, which enables better identification of isoforms. In the simulations parameterized by two real RNA-Seq data sets, more than 77% expressed isoforms are correctly identified and quantified. Then, we apply NSMAP on two RNA-Seq data sets of myelodysplastic syndromes (MDS) samples and one normal sample in order to identify differentially expressed known and novel isoforms in MDS disease.

**Conclusions:**

NSMAP provides a good strategy to identify and quantify novel isoforms without the knowledge of annotated reference genome which can further realize the potential of RNA-Seq technique in transcriptome analysis. NSMAP package is freely available at https://sites.google.com/site/nsmapforrnaseq.

## Background

More than 90% of human genes [1,2] are estimated to be alternatively spliced which leads a single gene to produce multiple proteins with distinct functions and is implicated in many diseases including cancer [3]. In recent years, there is an increasing interest in the use of alternative splicing in developing diagnostic tools and in identifying new therapeutic targets [4]. Microarrays have been widely used to analyze alternative isoforms by combining exon arrays and exon junction arrays to quantify isoform level expression indexes [5,6]. However, array based techniques are encountering several fundamental problems such as cross hybridization and weak signals in junction probes which are difficult to overcome [7]. Ultra high-throughput sequencing of RNA has been developed as an approach for transcriptome analysis in several different species and has offered an attractive approach to measure transcription in a comprehensive manner. RNA-Seq allows the direct detection of alternative splicing using the reads mapped at splice junctions including the novel splicing without the annotation information. Genome-wide measurements of transcriptomes are increasingly done by RNA-Seq which provides a far more precise measurement of expression levels of isoforms than other methods [8].

The rapidly-developing RNA-Seq techniques require substantial algorithmic advances. Several tools and strategies have been proposed to deal with the complex bioinformatics analysis of RNA-Seq [9-12]. Pepke *et al. *[13] provided a comprehensive and up-to-data review of multi-layered analyses of RNA-Seq data. Mortazavi *et al. *[10] proposed to quantify the gene level expression of a transcript as Reads Per Kilobase per Million mapped reads (RPKM). Further, Jiang and Wong [14] presented a statistical model to describe how the isoform expression levels were calculated from the number of reads mapped to the annotated exons of a gene. Meanwhile, Bohnert *et al. *[15] also proposed rQuant to determine the abundances for each annotated isoforms by minimizing the deviation of the observation from the expected position-wide read coverage. All these methods assumed that the number and structures of isoforms of each gene are known from the reference genome. However, as Jiang and Wong [14] pointed out, the isoform level annotation is very incomplete due to the complexity of the transcriptome and the limitations of previous experimental approaches. To address this issue, Trapnell *et al. *proposed Cufflinks [16] to identify transcripts as well as to estimate the expression levels of identified transcripts from mapped reads without annotation information. In essence, Cufflinks constructs a covering relation on the read alignments from TopHat [12], and find a minimum path cover on the directed acyclic graph for the relation based on Dilworth's Theorem [17] to construct a parsimonious set of transcripts. After that, the expression levels of those constructed transcripts are estimated using established known isoform expression estimation methods [9,14]. Therefore, the construction of transcripts in Cufflinks is independent of the expression level estimation. However, the construction of expressed transcripts and expression level estimation are highly associated. We argue that the determination of parsimonious set of expressed transcripts and expression level estimation should be implemented jointly. Though Scripture [18] can also detect the novel isoforms, the issue of parsimonious expressed isoforms is not addressed. We also notice that Feng et al. proposed IsoInfer [19] to identify isoforms using the detected junctions. The candidate isoforms were constructed by combining the putative exons followed by selecting a minimum best subset from all the enumerated subsets of the candidate isoforms which can explain the observation best. However, enumerating all possible subsets of the candidate isoform set with a given size is often infeasible. IsoInfer decomposes the large putative exon set into subsets to address this issue which introduces more parameters.

Here, we put forward NSMAP to infer the structures of isoforms as well as to estimate the expression levels simultaneously. First, the exons are constructed based on the detected splicing junctions from RNA-Seq data using TopHat. All the possible isoforms are enumerated by combination of those detected exons. Then NSMAP is applied to identify the true expressed isoforms from the large candidate isoform set as well as to estimate the expression levels with a sparsity control term to restrict the number of expressed isoforms. The assumption behind this sparsity term is that only as few isoforms as possible should be selected to best explain the observed number of reads falling on each exon of a gene. Finally, a model selection step is conducted to select the solution which compromises the fitting of the observation and the number of expressed isoforms best. In summary, our algorithm allows for discovering the structures of the expressed isoforms of a given gene and for estimating the concentration of each spliced isoform simultaneously without the annotated isoform information, which makes the identification of new, previously unknown, alternatively spliced isoforms possible. This study will help RNA-Seq, a next generation sequencing technology, advance to its full potential in comprehensive transcriptome analysis.

## Results

### Data set

We test NSMAP on two simulations with simulated expression levels derived from two publicly available mouse RNA-Seq as described in [10]. We also apply NSMAP on three real in-house RNA-Seq data sets of myelodysplastic syndromes (MDS) transcriptome analysis to identify isoforms including novel ones featured in MDS disease. MDS are a diverse collection of hematological conditions united by ineffective production of myeloid blood cells and risk of transformation to acute myelogenous leukemia whose frequency and incidence are increasing in the US population [20]. In our application, cryopreserved marrow cells and paraffin embedded marrow clot sections and marrow core biopsies from 2 MDS patients as well as 1 age-matched control sample are being studied by Dr. Jeff Chang's lab at the Methodist Hospital. These MDS patients have been thoroughly evaluated for clinical/morphologic/immunophenotypic data and characterized clinically by transfusion dependency and pathologically by significant dysplasia, increased blasts, and immunophenotypic aberrancy. The control sample are obtained from patients without cytopenias (> 60 years old). We specifically selected these controls to be age-matched for the MDSs population to control for the possibility of aging-related changes in the expression profile mRNA of hematopoietic cells. Then we apply the RNA-Seq protocol to sequence our samples. We sequenced the two MDS clinical samples and one normal sample using Illumina Genome Analyzer II. There are around 40 million single-end reads with read length 76 bp for each sample.

### Algorithm Summary

NSMAP comprise four consecutive steps, starting with junction detection and reads mapping using TopHat [12] and followed by the candidate isoforms construction, expressed isoform identification and expression level estimation along whole regularization path and model selection to select the best solution from the whole solution path. Short reads alignment is the first step in understanding next-generation sequencing data and many free alignment software packages are available [21,22]. Here we use TopHat to perform the alignment task which can detect the junctions and map a massive amount of reads to the whole genome flexibly and efficiently. The reference genome sequences are downloaded from UCSC genome database [23]. After read alignment, the next step is to generate the candidate isoforms according to the alignment and splice junctions obtained from TopHat.

#### Candidate isoforms construction

**Figure 1:**
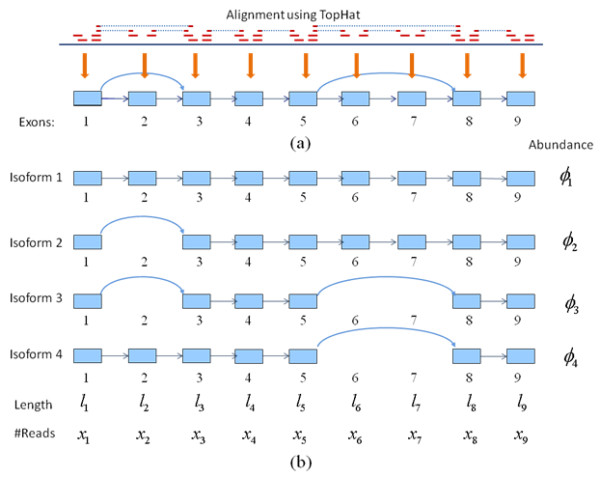
**The construction of exons and isoforms from the alignment result of TopHat**. (a) 9 exons are constructed from the alignment result of TopHat where the dotted line means a junction detected by a read and one arrow line represents a detected junction between two exons; (b) four possible isoforms constructed from source exon 1 to sink exon 9 using path finding algorithms from graph theory where *l*, *x *and *ϕ *indicate the exon length, the number of reads falling on this exon and the unknown abundance of each isoform, respectively.

Based on the alignment results and detected junctions from TopHat, the exons can be constructed from segments whose two ends have been detected as junction points. For example, Figure 1(a) mimics a gene with nine exons constructed from the detected junctions in a gene region. The isoforms are formed from combination of those exons. Each rectangle represents an exon and arrow line indicates that there is a splice junction detected between the two exons. This can be interpreted as a directed graph with each exon as nodes. Each candidate isoform can be generated by finding a path between two nodes on this graph. For instance, Figure 1(b) displays the possible isoforms with exon 1 and 9 as source and sink respectively. There are total four paths connecting exon 1 and 9 as indicated in Figure 1.

#### Isoform Expression level estimation

Given the candidate isoforms constructed from last step, we need to select the expressed isoforms from this candidate set as well as to estimate the express level of selected isoforms. We adapted the expression level estimation framework of Jiang and Wong [14] by incorporating a sparsity regularization term. For a gene g, suppose it has *m *exons with lengths [*L *= *l*_1_,...,*l*_*m*_] and *n *isoforms with expression Φ = [*ϕ*_1_,...,*ϕ*_*n*_]. Assuming each exon can be either included in an isoform or not, we have a set of observations , where  is an index set of events which we are interested in. Each observation *X*_*s *_∈ **X **is a random variable representing the number of reads falling into a certain region of interest in gene g. For example, reads falling into certain exon or exon-exon junction.

The natural statistical model of count data is the Poisson distribution. Each *X*_*s *_∈ **X **follows Poisson distribution with a parameter *λ *which is the expected count or the mean parameter of the Poisson distribution. For instance, the *λ *for the number of reads falling into exon *j *is , where *N *is the total number of mapped reads and *c*_*ij *_is 1 if isoform *i *contained exon *j *and 0 otherwise. For a exon-exon junction event, the *λ *is , where *l *is the length of the junction region, *j *and *k *are indexes of the two exons involved in the junction being investigated.

In general, *λ*_*s *_is a linear function of *ϕ*_1_,*ϕ*2,..,*ϕ*_*n*_, i.e.  where *a*_*si *_is a known coefficient. The likelihood function is

Maximum likelihood estimation (MLE) method can be used to estimate Φ by maximizing above likelihood function [14]. However, in the work of Jiang and Wong [14], the structure of individual isoform is fetched from the reference genome where only a few of isoforms are known for each gene. In our application, the structures of expressed isoforms are unknown which need to be selected from the large candidate isoform set where each isoform is formed by combination of detected exons. To do this, the most important prior information is arguably sparsity; that is, characteristic pattern of each gene in cells are expressed by very few isoforms, even though the total number of possible combinations of exons in a gene can be large. Utilization of this sparsity information is crucial to the success of finding the most relevant isoforms which explain the observed reads best, in the face of "insufficient" data and/or data uncertainty [24]. To incorporate this sparsity to our model, we propose a prior Laplace distribution on Φ as below:

Laplace distribution assigns higher probability at zero than Gaussian distribution to produce sparser solution and has been widely used to encode sparseness prior [25]. Hence we can employ maximum a posteriori (MAP) instead of maximum likelihood to estimate Φ:

Taking logarithm on the above equation, we get

Keep in mind that the expression of each isoform cannot be negative, that is *ϕ*_*i *_≥ 0 (*ϕ*_*i *_∈ Φ), *F*(Φ) can be rewritten as:

where *ϕ*_*i *_≥ 0 for *i *= 1,...,*S *and *S *is the number of events with which we are interested. Maximizing *F*(Φ) with respect to Φ is equivalent to minimizing

To that end, the optimization problem is summarized as:(1)

We call this Nonnegativity and Sparsity constrained MAP model as NSMAP. Laplace prior distribution has a *L*_1 _norm term to impose the sparsity. In some cases of our experiment, we find it may not work well in the identification of the correct expressed isoforms due to the similarities between the isoforms. The ideal sparsity constraint is the *L*_0 _regularization, defined as the number of non-zero entries in a vector. Unfortunately, the computation of *L*_0 _regularization is intractable to solve. *L*_1 _norm is popular because of its intrinsic convex property. Some recent works suggest that non-convex regularizations such as *L*_*p *_regularization with 0 < p < 1 have better performances in parameter selection and sparse signal construction [26] than *L*_1 _regularization. This inspires us to use a stronger sparsity constraint with *L*_*p*_, 0 <*p *< 1 norm to solve our problem. We will implement a simulation study to compare the performances of sparsity regularizations with different *p*. In our experiment, we set *p *= 0.5. So the objective function is finally given by:(2)

where  and .

Then the expression levels of all the candidate isoforms can be obtained by optimizing Equation (1) with a given *t*. When *t *→ +∞, the solution  equals zero without expressed isoforms. With the decreasing of *t*, some elements of  will be non-zero to become expressed isoform. So we have to select an optimal *t*. Before doing this, we will calculate the solution path which consists of solutions corresponding to different values of *t*. BLasso [27] is adapted to approximate this solution path efficiently (see Methods).

#### Model selection from solution path

After getting the solution path which consists of solutions corresponding to different values of *t*, we have to select the best solution (model) from this solution path which makes a good balance between the number of expressed isoforms and the fitting function  in equation (2). The solution path will first be grouped into subsets with increasing model size according to the number of expressed isoforms of each solution (the number of positive components of ). Here the model size means the number of expressed isoforms. Then the solution with minimal  in each group is selected as the best solution for each model size. Starting from model size 1, we compare the  of current model size with the next model size which have one more expressed isoforms. If  is significantly improved by the larger model size, the model with larger model size will be updated to the current model and compared with the remaining models with larger model size than the updated model. Otherwise the solution  corresponding to the current model size is selected as the final solution. In this way, the solution with smaller number of expressed isoforms will be selected preferably. See the Methods for more detail.

### Simulation on the whole genome with different *L*_*p *_norm in equation (2)

In the absence of RNA-Seq data from samples for which we have ground truth isoform quantities, we conduct simulations to validate our method and evaluate its performance in terms of isoform identification and expression levels estimation with *p *= 1 and *p *= 0.5.

We first derive expression level of each isoform from the mouse brain and liver RNA-Seq data sets described in [10] using the Poisson model of [14] with the mouse UCSC Genes as the annotated reference genome database. Those derived expression levels are employed as the ground truth to perform the following simulations. Which exon or splice region the read will fall on is determined by uniformly sampling proportional to the simulated ground truth RPKM and the same mapped reads number of each gene in the real data. After uniformly sampling and counting reads number, we can identify isoform structures and their concentrations from the simulated RNA-Seq reads data and evaluate the performance of NSMAP with the simulated ground truth.

We only consider isoforms with expression levels no less than 1 RPKM as expressed isoforms. In this simulation, constructed isoforms containing more than half of the total exons are eligible as the candidate isoforms. The accuracy of NSMAP largely depends on the critical step of candidate isoform generation. So, we check whether our candidate isoforms generated from the exon reads and exon junctions can cover the true expressed isoforms. From Table 1 we see that more than 96% of expressed isoforms in the two data sets are included in the candidate isoforms generated by our strategy which finds paths on a graph constructed from exons and splice junctions.

**Table 1:** The percentage of expressed isoforms with RPKM ≥1 included in the candidate isoform set.

Tissue	Total number of expressed isoforms	Percentage of expressed isoforms included in candidate isoforms
Brain	14,154	96.5%

Liver	10,583	96.1%

We further compute the fraction of isoforms for which the estimates are significantly consistent with the simulated ground truth (percent error = ). We refer to this statistic as the *positive fraction *[9]. Given the positive isoforms which are within the 5% deviation from the ground truths, overall specificities of the two simulations are also calculated by setting the truly expressed isoforms with RPKM no less than 1 as positive isoforms and the false reported isoforms by NSMAP and truly expressed isoforms with RPKM < 1 as negative isoforms.

The results are summarized in Table 2. We can see that NSMAP achieved near 80% overall positive fraction in both simulations. And highly expressed isoforms (with high RPKM) have high positive fraction. While for isoforms with low RPKM, the accuracy is not very good because the number of reads falling on this isoform is small which may not be sufficient to capture the exons and splice junctions to form this isoform. This phenomenon is consistent with other studies [28] that the estimation on the low abundance isoform is not very accurate.

**Table 2:** Positive fraction and specificity of the estimation results of NSMAP on the two simulated data sets.

Isoform expressions in RPKM						
**Tissue**	**RPKM**	**[1,10)**	**[10,10**^**2**^**)**	**[10**^**2**^**,10**^**3**^**)**	**[10**^**3**^**,10**^**4**^**)**	**Total**

Brain *p *= 1/2	Number of isoforms	7,730	5,630	767	27	14,151
	
	Positive fraction	67.9%	88.2%	95.4%	100.0%	77.5%
	
	Specificity					83.6%

*p = *1	Positive fraction	67.8%	87.9%	95.3%	100.0%	77.3%
	
	Specificity					83.3%

Liver *p *= 1/2	Number of isoforms	6,470	3,390	661	62	10,583
	
	Positive fraction	70.0%	89.8%	97.1%	100.0%	78.2%
	
	Specificity					90.3%

*p = *1	Positive fraction	69.5%	89.7%	97.1%	100.0%	77.9%
	
	Specificity					89.8%

Comparing the performances of NSMAP with *p *= 0.5 and *p *= 1, we notice that *L*_*p *_norm with *p *= 0.5 provides better results than *p *= 1 in the simulations. To explain this observation, we conduct the following experiment and give a mathematical interpretation of the two *L*_*p *_norms.

### Demonstrations of features of *L*_*p *_norm with *p *= 0.5 and *p *= 1

**Figure 2:**
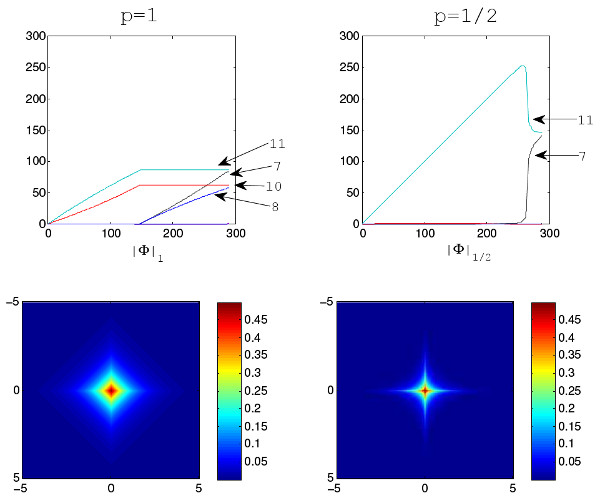
**Simulation experiments with different *L***_***p***_** norm**. Upper Row: Solution paths generated by BLasso for NSMAP with different *p *norm on the simulated data on the gene Eif5a. The Y-axis is the estimated isoform concentrations ranging [0,300]. The X-axis for two figures in upper panel is . Lower Row: The probability density functions of Laplace distribution and a Laplace-like distribution with *L*_1/2 _norm *P*(**x**)∝*exp*(-||**x**||_1/2_); both of them have unit variances.

First, we exemplify our observation in the above simulation that *L*_*p *_norm with *p *= 0.5 is better than *p *= 1 through a simulation using the structure of gene Eif5a. In the UCSC genome annotation, gene Eif5a has 14 exons and 9 known isoforms. We set both RPKM expression levels of the fifth and sixth isoforms as 150 because the two isoforms express most from the estimation using the method of [14] on the mouse brain and liver RNA-Seq data in [10]. The simulation is performed based on the expression levels and structures of the two expressed isoforms under the uniform distribution for reads. Based on the simulation data, total 71 candidate isoforms is constructed. The indexes of the two truly expressed isoforms in the candidate set are 7 and 11, respectively. From the upper row of Figure 2, we can see that the two expressed isoforms are selected correctly when *p *= 1/2, while in the case of *p *= 1, additional two false unexpressed isoforms (with indices of 8 and 10) are also selected.

Then, the lower row of Figure 2 shows the mathematical properties of the two norms. *L*_1/2 _norm regularization has more similar sparsity property with *L*_0 _norm than *L*_1 _norm because the points with high probabilities in the Laplace-like distribution with *L*_1/2 _norm are more focusing around the axes than those in the Laplace distribution. This means *L*_1/2 _norm regularization imposed stronger sparsity than *L*_1 _norm. The simulation depicts the stronger sparsity constraint by setting *p *= 1/2 is superior to Laplace priori distribution with *p *= 1 in our application. In the following experiments, we select the *L*_*p *_norm with *p *= 1/2.

### Comparison with IsoInfer on two RNA-Seq data sets of MDS samples

Here we compare NSMAP with IsoInfer which have similar ideas in isoform construction from putative exons and minimum expressed isoform set selection. However, IsoInfer selects a subset as expressed isoforms from candidate isoform set by enumerating all the possible subsets of the candidate isoform set. In NSMAP, the selection of expressed isoforms is embedded into the isoform expression level estimation framework by incorporating a sparsity control term.

A transcript can be constructed from all the exon-intron boundaries as well as the transcription start site (TSS) and poly-A site (PAS) of an isoform. The exon-intron boundaries can be inferred from RNA-Seq using alternative splicing detection tool, such as TopHat and SpliceMap [29]. The TSS and PAS represent the start and end expressed segments of a transcript, respectively. Theoretically, any expressed segments can be the TSS or PAS which will introduce many false short isoforms to make isoform inference difficult. We prefer to retrieve the TSS-PAS from the UCSC known isoform table as the starts and ends of predicted transcripts and to identify isoforms within the regions of known genes whose functions and pathways are intensively studied. IsoInfer (version 0.9.1) and NSMAP will use those TSS-PAS and the detected junctions using TopHat to infer the expressed isoforms from RNA-Seq. Because it is infeasible to validate all the predicted isoforms, we evaluate the two methods by comparing their predictions with UCSC known isoform data set. The performance of the method is measured by sensitivity and precision. Here we use hg19 known human isoforms data set downloaded from UCSC which contains 77,614 transcripts. A known isoform is identified if it is in the prediction result of a method. Sensitivity is defined as the number of identified isoforms divided by the number of all known isoforms from UCSC data base. Precision is defined as the number of identified known isoforms divided by the number of predicted isoforms by the method.

**Figure 3:**
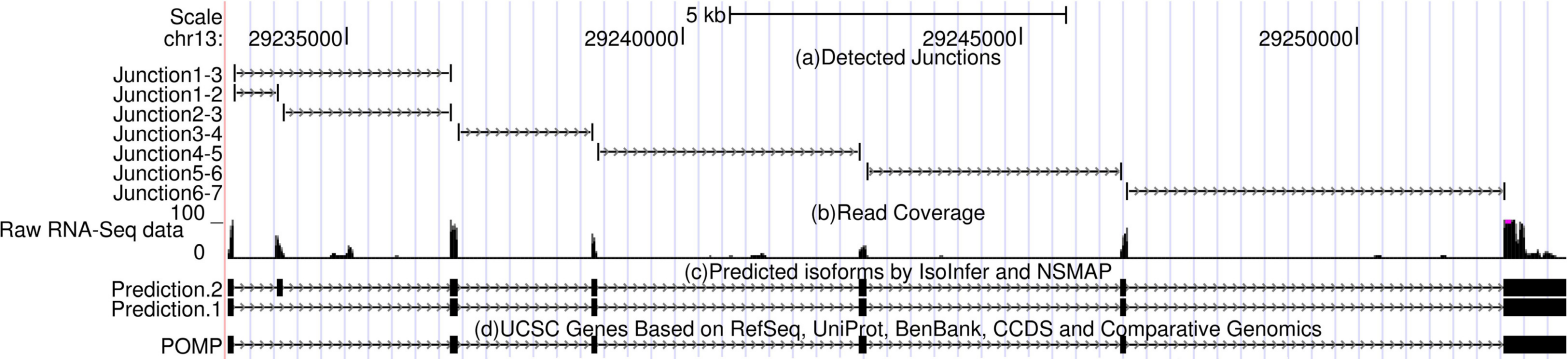
**The prediction results of NSMAP and IsoInfer on a genome region based on MDS sample 1**. (a)Detected Junctions using TopHat; (b) Read coverage of the real data on this region (chr13:29233240-29253091); (c) Two isoforms predicted by NSMAP and IsoInfer. One is novel and another one is annotated by UCSC known isoforms (d) The known isoform (gene POMP) on this region.

Table 3 summarizes the results of the two methods as well as their performances. Though NSMAP predicted less known isoforms than IsoInfer, NSMAP achieves higher precision than IsoInfer. Most of the known isoforms which are predicted by both methods are common. For example, in MDS sample 1, 857 of 1128 known isoforms predicted by IsoInfer are also identified by NSMAP. This is reasonable because NSMAP and IsoInfer have similar ideas in isoform construction from putative exons and minimum expressed isoform set selection. Figure 3 shows a known and a novel isoform predicted by both NSMAP and IsoInfer where the novel isoform is very consistent with the read coverage from RNA-seq data.

**Table 3:** The performance of IsoInfer and NSMAP on two RNA-Seq data of MDS samples by comparing the results with the UCSC known isoforms.

Samples	MDS 1		MDS 2	
#Mapped reads	18,729,721		22,436,651	

Methods	IsoInfer	NSMAP	IsoInfer	NSMAP

#Predicted isoforms	10,695	9,772	12,466	10,185

#Identified known isoforms	1,128	1,078	1,632	1,469

#Common known isoforms predicted by both methods	857		1103	

Sensitivity	1.5%	1.5%	2.1%	2.0%

Precision	10.5%	11.0%	13.1%	14.4%

However, IsoInfer selects a subset as expressed isoforms from candidate isoform set by evaluating all the possible subsets of the candidate isoforms. In NSMAP, the selection of expressed isoforms is embedded into the isoform expression level estimation framework by incorporating a sparsity control term. In this way, the selection of expressed isoforms is automatic and more efficient than testing all the possible sub sets of the candidate isoforms.

We also notice that more known isoforms are predicted in MDS sample 2 than MDS sample 1, because there are more reads are mapped in MDS sample 2. This observation tells us that deeper sequencing will improve the performances of IsoInfer and NSMAP.

Here, the sensitivities of the two methods are very low. The reason is that we compare the predicted isoforms with the large UCSC known isoform set. Some of the UCSC known isoforms may not express in the sample. So the effective sensitivities will be larger than those numbers. This comparison against UCSC known isoform data set does not mean all the predicted isoforms without annotation are false. Especially those predicted novel isoforms with high RPKM are promising to be true novel isoforms. For example, if we select the predicted isoforms with expression level larger than 100 RPKM, in MDS sample 1 and 2, 37 out of 67 and 53 out of 105 predicted isoforms by NSMAP will be annotated in the UCSC known isoforms table. And the lowly expressed isoforms have higher probability to be false positive or artifacts due to the insufficient reads for capturing the true structure of an isoform. So we need to set an expression level threshold to refine the predicted isoforms. This issue is addressed in the following section.

### Example of identified isoforms using NSMAP and Expression Level Threshold Selection

**Figure 4:**
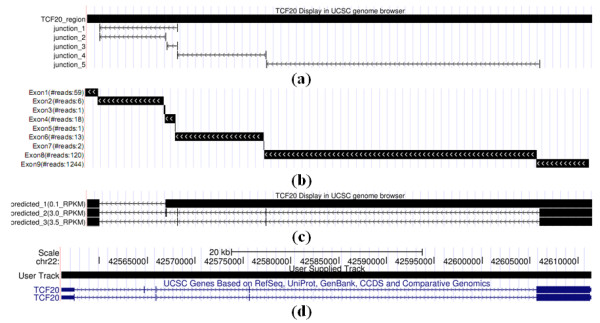
**Take gene TCF20 as an example to demonstrate the procedure and prediction result of NSMAP**. (a)The region of gene TCF20 and the detected splicing junctions using Tophat. (b) The constructed exons based on the detected splicing junctions for gene TCF20. The number in the parentheses of the each left label is the number of reads falling on the corresponding exon. (c) NSMAP predicts three isoforms for TCF20 with expression levels in the parentheses. (d) The two annotated isoforms of gene TCF20 in the UCSC reference genome.

Here we exemplify isoforms of a gene TCF20 estimated by NSMAP. TCF20 encodes protein transcription factor 20 which localizes to the nucleus and displays DNA-binding and transactivation activities. TCF20 has five exons and alternative splicing results in two known transcript variants encoding different isoforms in the UCSC reference genome (Figure 4(d)). The RNA-Seq data is first processed by TopHat to detect the splicing junctions. Figure 4(a) shows the splicing junctions of TCF20 on MDS sample 1 detected by TopHat. Then nine exons are constructed based on detected junctions in Figure 4(b). NSMAP predicts three isoforms with expression level 0.1, 3.0 and 3.5 respectively (Figure 4(c)). Compared with the annotated isoforms of TCF20 (Figure 4(d)), we confirm that the top two highly expressed predictions are the same with the annotations. This example demonstrates the ability of NSMAP in isoform identification.

**Figure 5:**
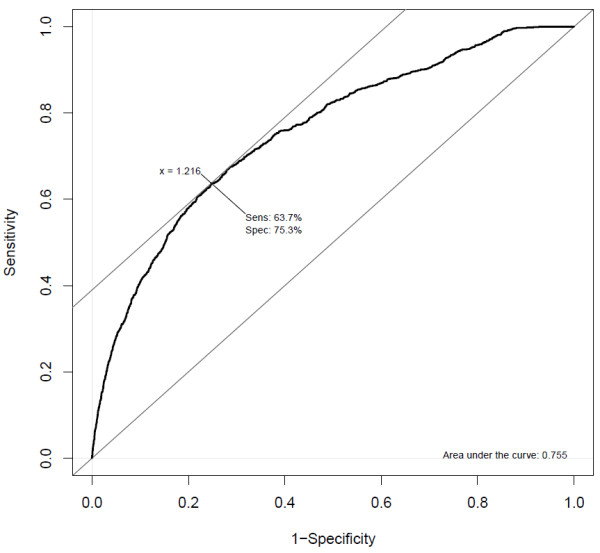
**ROC curve of the prediction of NSMAP on MDS sample 1 and the selected expression level threshold**.

However, NSMAP still predicts an additional lowly expressed isoform (0.1 RPKM) which is not annotated in the UCSC known isoform. Higher expression level of an isoform means more reads will be sampled from this isoform to capture the structure of this isoform. So the predicted isoforms with low expression levels have more probability to be artifacts. We need an expression level threshold T to exclude those lowly expressed isoforms. The selection of T is a trade-off between sensitivity and specificity. The smaller the value T is, the higher sensitivity but lower specificity the result will achieve. Because there is no ground truth for the predicted novel isoforms, we set the predicted isoforms which are annotated by the UCSC known isoforms as positive, others as negative. To determine this value, we explore the ROC curve of the prediction result of MDS sample 1 where the TCF20 in Figure 4 comes from. The ROC curve and selected threshold of the prediction result of NSMAP on the MDS sample 1 are depicted in Figure 5. The optimal threshold T in this ROC curve is 1.216. The intuition of this optimal threshold is that at this point, the increase rate of sensitivity equals to the increase rate of (1-Specificity). When the threshold is infinite, both sensitivity and (1- Specificity) are 0. With the decrease of threshold T, both sensitivity and (1-specificity) will increase. But the increase rate of sensitivity is larger than that of (1-specificity) until T reduces to the point 1.216. After that point, the increase rate of (1-specificity) will be larger than sensitivity. (1-specificity) actually is the false positive rate. We prefer higher sensitivity and lower false positive rate. So we select this point as the optimal point.

In the predicted isoforms of gene TCF20 in Figure 4, the two annotated isoforms have expression levels 3.0 and 3.5, respectively, which are above the selected threshold T = 1.216. While the expression level of the un-annotated isoform is 0.1 which is obviously under this threshold. In this way, we perform a further refinement of the prediction result. In the following real data analysis, we only consider NSMAP predicted isoforms which are larger than the optimal expression level threshold in each sample.

### Clinical MDS sequencing data analysis

The goal is to use our NSMAP to identify known and novel isoforms which may be related with MDS. We apply NSMAP on the alignment results of the three data sets from TopHat to identify the expressed isoforms and their expression levels. The predicted isoforms are compared with the UCSC annotated reference genes to distinguish the known and novel isoforms.

For the known isoforms, we select the differentially expressed isoforms with fold change greater than 2 on both disease samples compared with the control sample. We also select MDS featured novel isoforms which are detected in both MDS samples but are absent in the normal sample. Finally, we get total 785 differentially expressed known isoforms and 128 novel isoforms with RPKM over 5. The two selected isoform sets were fed into Ingenuity Pathways Analysis (IPA) [30] respectively. IPA is a database search tool for finding function and pathway for specific biological states. Here we used IPA to explore the pathways enriched in the two selected isoform sets. The top 4 enriched canonical pathways of the two analyses are listed in Table 4 where mitochondrial dysfunction is both enriched in the known and novel differentially expressed isoform sets which is known to closely related with MDS [31]. The Oxidative phosphorylation and mitochondrial dysfunction are enriched by both differentially expressed isoform sets which mean the novel differentially expressed isoforms have similar biological functions with the differentially expressed known isoforms. That observation indicates the prediction of novel isoforms is consistent with the known isoforms.

**Table 4:** The top 4 enriched canonical pathways from Ingenuity Pathways Analysis of the differentially expressed known isoforms and novel isoforms.

Differentially Expressed known isoforms	Differentially expressed novel isoforms
Granzyme A signalling	Mitochondrial dysfunction

Oxidative phosphorylation	Oxidative phosphorylation

Mitochondrial dysfunction	Methane metabolism

cAMP-mediated signaling	Protein ubiquitination pathway

## Discussion

Through simulations that closely modelled real data, we confirm our method's effectiveness for experiments in both mouse brain and liver RNA-Seq data. We also compare NSMAP with IsoInfer to show that NSMAP has comparable performance in identifying known isoforms from RNA-Seq reads. Finally, we apply NSMAP on our MDS RNA-Seq data analysis and find some differentially expressed known isoforms and novel isoform candidates which involve in some MDS related pathways.

Recently, Lacroix *et al. *[32] showed that unique solution cannot be guaranteed theoretically in isoform identification from short sequence reads. For example, in our case, all possible transcript isoforms are enumerated according to the detected junction reads. Among these isoforms, one truly expressed isoform may be linear combinations of the other isoforms in terms of exon arrangements. Then the solution of this case is not unique. The assumption of NSMAP to address this issue is that the solution which employs as few expressed isoforms as possible to explain the most observation is preferred. Though this assumption is identical to the assumption made by Cufflinks, the implementation of this assumption in NSMAP is totally different from Cufflinks. Cufflinks constructed a parsimonious set of transcripts followed by the expression level estimations of those constructed transcripts using established expression level estimation model. However, NSMAP enumerates all the possible isoforms formed by the combinations of identified putative exons from TopHat and incorporates a prior distribution into the expression level estimation model to control the number of expressed isoforms. That means the identification of expressed isoforms and the expression level estimations of those identified isoforms are done jointly in NSMAP.

Paired-end sequencing can dramatically improve the accuracy of isoform level expression estimation which is becoming ubiquitous. Recently, Salzman *et al. *[33] proposed "insert length model" to extend Jiang and Wong's single-end sequencing work [14] to paired-end sequencing analysis by modeling the insert length distribution. So we can use this idea to handle paired-end sequencing data in our current framework.

Paired-end sequencing can dramatically improve the accuracy of isoform level expression estimation which is becoming ubiquitous. In paired end sequencing, only the fragments in a specified range will be selected. Several papers have used this information by modeling the fragment length distribution to improve isoform deconvolution problem [16,34]. Salzman et al. [33] proposed "insert length model" to extend Jiang and Wong's single-end sequencing work [14] to paired-end sequencing analysis by modeling the fragment length distribution. So we can use this idea to handle paired-end sequencing data in our current framework. In paired-end sequencing, Salzman et al. defines *a*_*si *_= *q*(*f*_*si*_)*N *for an event *s *where the mate reads are mapped into two specified positions on genome. Here *f*_*si *_is the length of corresponding fragment on the *i*-th transcript and *q*(*f*) is the probability of observing a fragment with length *f*. In practice, *q*(*f*) can be approximated by the empirical probability mass function computed from all the mapped paired-end reads. In order to reduce the number of events, the minimal sufficient statistics is used to group the events into minimal categories for computational purpose. In this way, we can incorporate the paired-end information into our model by redefining *a*_*si *_to address paired-end sequencing data.

Currently, NSMAP uses the TSS and PAS retrieved from UCSC known isoforms. We will extend it to identify TSS and PAS from RNA-seq by the following scheme. If the start point of a putative exon is not a junction point, this putative exon can be regarded as TSS. And if the end point of a putative exon is not a junction point, this putative exon can be regarded as PAS. Here junction point means this point is the start or end of a splicing junction.

As our primary motivation is to design a method to identify the isoform structure without annotated reference isoform genome, the usefulness of NSMAP is largely dependent on the expression levels of true isoforms and splicing junction detection. We believe that the accuracy of this approach will increase significantly as the sequencing technology evolves such as paired-end sequencing technique and generates longer sequences with less noise and higher throughput.

## Conclusions

In this paper, we propose a statistical model NSMAP for RNA-Seq data analysis which can be used to identify and quantify isoforms simultaneously without isoform annotations from reference genome.

## Methods

### Implementation

We must select a particular value of *t *at which the estimation is optimal. Before that, the solutions corresponding to different values of *t *should be calculated. Efron *et al. *[35] proposed an efficient algorithm LARS to determine the exact piecewise linear coefficient paths for the lasso. Unlike lasso, the path of our solution is not piecewise linear. To address this non-piecewise solution issue, we modify the BLasso [27] to get the solutions corresponding to different *t*. The basic ideal of BLasso is to correct the forward stage-wise boosting algorithm by allowing backward steps whenever a stop in forward stage-wise boosting fitting deviates from that of the lasso.

Optimization: Generalized BLasso for NSMAP:

• Step 1(Initialization) Given a small step-size constant *ε *> 0 and a small tolerance parameter *ξ *≥ 0, take an initial forward step on *L*(Φ^(0)^) in equation (2). We define Φ^(0)^≜*m***1**_*j*_.

Herein  because of the non-negative constraint and **1**_*j *_is a *n*-dimensional standard basis vector with all 0's except for a 1 in the *j *th entry. So  where *L*(Φ) is defined in equation (2). Then calculate the initial regularization parameter

We use **I**_*A *_to represent the active index set. Set the initial active set  and *k *= 0.

• Step 2 (Steepest descent step). Find the steepest coordinate descent direction on *J*(Φ;**t**) in equation (2):

Take the step if it leads to a decrease of moderate size *ξ *in the objective function *J*(Φ;**t**):

If 

then 

Otherwise, adjust  to minimize *L*(Φ) in equation (2) and recalculate the regularization parameter *t*:

Update  whose elements are the indexes of the positive entries of .

• Step 3 (iteration). Increase *k *by one and repeat Step 2 until *t*^(^^*k*^^) ^≤ 0.

Computationally, BLasso takes roughly *O*(1/*ε*) steps to produce the whole path [27]. The actual computation complexity depends on the actual objective function and minimization method used in each step when calculating . In the following experiments, ∈ is set as 0.1 and *ξ *= 1*e *- 10.

### Model selection and expression level estimation

**Figure 6:**
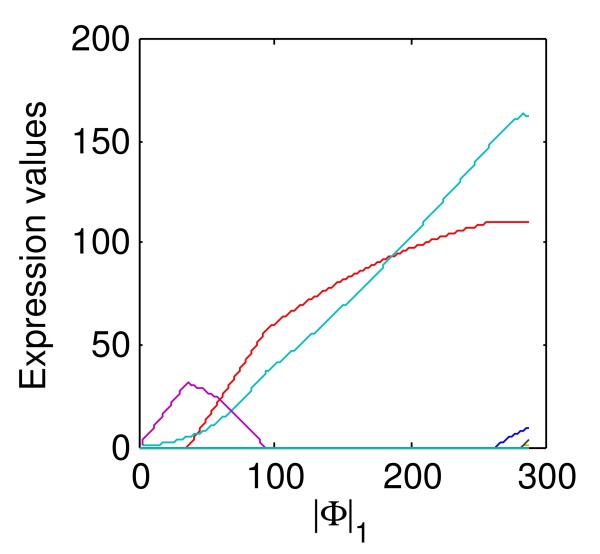
**An example of solution path {Φ^(*k*) ^| *k *= 0,1,...,*K*} along the X axis**. Each curve represents the expression level of one candidate isoform during the iterations. The solution path is plotted from left to right along the X axis. This figure shows the number of expressed isoforms varies along the solution path.

A sequence of solutions  corresponded to the decreasing *t *during iterations where *K *is the number of iterations. Each regularization parameter *t*^(^^*k*^^) ^has a solution with several isoforms  selected as active. There is only one selected isoform in  when *t *is on its largest value *t*^(0)^. With the decreasing of *t*, more isoforms are selected into the active set and the number of expressed isoforms varies as shown in Figure 6. We should select the best model from these solutions which can explain as more observations as possible using as few expressed isoforms as possible. The number of expressed isoforms of a solution equals with the number of positive elements in . In order to control the model size, we group the sequential solutions  into  according to the number of positive elements in each solution , where  is used to count the number of positive elements in solution  and H_*h *_is a subset of . The number of expressed isoforms of each solution in H_*h *_equals *h*.

We first select the best solution within each group H_*h*_. Because the model size within each group is the same, the solution  whose  is minimal in group H_*h *_is selected as the best solution in this group.

Those best solutions  in each group are put into a set  ordered by the increasing number of expressed isoforms where M is the largest number of expressed isoforms in the solution path and  has one expressed isoform which is the best solution among the solutions with one expressed isoform. The final best solution is selected from  by likelihood ratio test (LRT) [36].

**Figure 7:**
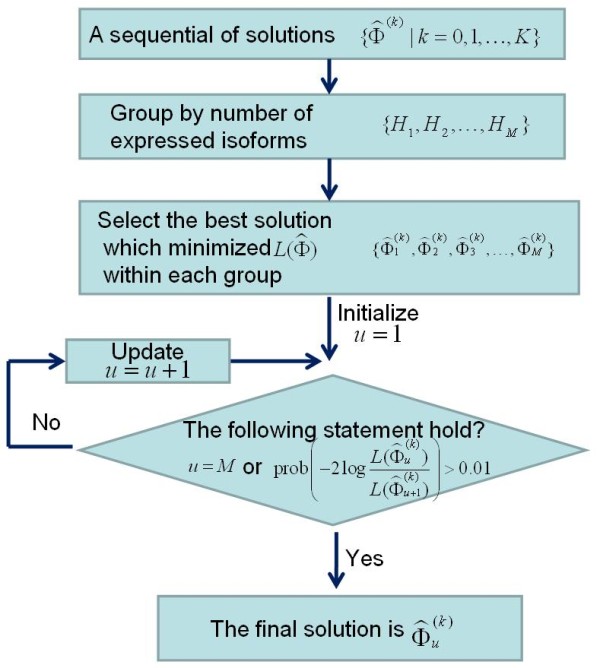
**The schematics of model selection from the solution path**.

Starting from the sparsest solution  with one isoform, we compare  with solution  which has two expressed isoforms to determine which solution is better and should thus be preferred using LRT by deriving the p-value of the obtained difference between  and . The test statistic of this difference is defined as . In most cases, the probability distribution of this test statistic *D *can be approximated by a chi-square distribution with *κ *= df(A)-df(B) degrees of freedom, where df(A) and df(B) are the degrees of freedom of models A and B respectively. That means *D *~ *χ*^2^(*κ *). In our case, the difference between the degrees of freedom of the two models are (df(A) - df(B)) = (2-1) = 1 and the p-value of *D *can be calculated according to *D *~ *χ*^2^(1 ). If prob(*D*) > 0.01 which means  is not significantly smaller than , we select  as our final best solution and stopped the searching. Otherwise,  and  are compared using LRT alike, and so on. The whole flowchart on model selection is summarized in Figure 7.

## Authors' contributions

ZX developed the method, implemented the code and drafted the manuscript. JW and CCC provided the MDS RNA-seq data and biological interpretation. XZ supervised this project and revised the draft manuscript. All authors read and approved the final document.
